# Morphological, phenotypical and molecular characterization of canine lymphomas with dual T- and B-cell markers expression

**DOI:** 10.3389/fvets.2025.1578425

**Published:** 2025-04-22

**Authors:** Giorgia Mezzalira, Valeria Martini, Francesca Abramo, Selina Iussich, Margherita Orlandi, Luca Pazzini, Barbara Banco, Anna Maria Rambaldi, Alessandro Bisognoso, Filippo Arena, Giulio Cocciolo, Michele Marino, Maria Massaro, Luca Aresu

**Affiliations:** ^1^MYLAV La Vallonea Veterinary Diagnostic Laboratory, Milan, Italy; ^2^Department of Veterinary Medicine and Animal Sciences, University of Milan, Lodi, Italy; ^3^Department of Veterinary Sciences, University of Pisa, Pisa, Italy; ^4^Department of Veterinary Sciences, University of Turin, Turin, Italy

**Keywords:** dog, lymphoma, immunohistochemistry, PARR, double phenotype

## Abstract

**Introduction:**

Recent investigations have identified rare, phenotypically complex lymphoma variants, including cases exhibiting concurrent expression of T- and B-cell markers. These atypical presentations suggest perturbations in lymphoid differentiation programs or clonal evolution, necessitating more sophisticated diagnostic approaches. The concurrent expression of CD3 and CD20 in canine lymphomas represents a particularly compelling phenomenon documented across various anatomical sites. Molecular diagnostics, particularly PCR for Antigen Receptor Rearrangements (PARR), have provided crucial insights into these phenotypically complex cases, revealing concurrent clonal rearrangements of both TCRγ and IgH in selected cases, further challenging traditional classification paradigms.

**Methods:**

Here, we report 33 cases of canine double-positive lymphoma, retrieved with a retrospective analysis of the MyLAV Diagnostic Laboratory electronic database. Specifically, we report results of an integrated approach combining WHO-based morphological classification, comprehensive immunohistochemical immunophenotyping with T-cell (CD3 and CD5) and B-cell markers (CD20 and PAX5), and PARR analysis.

**Results:**

The skin, oral/nasal mucosa and mucocutaneous junction were the most commonly affected sites, accounting for 24 cases (72.7%). All cases stained positive for CD3 and CD20 (100%), 32 (97%) for CD5, and only 12 (36.4%) for PAX5. Aberrant cytoplasmic localization of CD20 was found in 29 (87.9%) cases. Molecular analysis revealed rearrangement signals of TCR gene in 23 of 33 cases (69.7%) and of CBmajor or CBminor gene in 9 (27.3%).

**Discussion:**

The findings emphasize that while immunohistochemistry remains a fundamental diagnostic tool, it may be insufficient in isolation for definitive lineage determination in these cases. PARR analysis emerges as an essential complementary technique for distinguishing between aberrant marker expression and true biphenotypic differentiation.

## 1 Introduction

Canine lymphoma, a prevalent hematological malignancy, demonstrates considerable morphological and immunophenotypic heterogeneity, manifesting with diverse clinical presentation and anatomical distributions (1). Classification systems adapted from human oncology, notably the World Health Organization (WHO), have been implemented in veterinary medicine to enhance diagnostic accuracy and prognostic assessment. This system integrates morphological evaluation, and immunohistochemistry (IHC) (2). Immunophenotyping plays a critical role in lymphoma subtype differentiation (3, 4). This distinction carries significant clinical implications, as T-cell lymphoma typically demonstrate more aggressive biological behavior, reduced survival time, and diminished chemotherapeutic responses compared to their B-cell counterparts (1). Recent investigations have identified rare, phenotypically complex lymphoma variants, including cases exhibiting concurrent expression of T- and B-cell markers, or lacking definitive lineage markers (null-cell phenotype) (5–8). These atypical presentations suggest perturbations in lymphoid differentiation programs or clonal evolution, necessitating more sophisticated diagnostic approaches (9, 10).

The concurrent expression of CD3 and CD20 in canine lymphomas represents a particularly compelling phenomenon documented across various anatomical sites. Nicoletti et al. (11) characterized a high grade nodal lymphoma in a mixed breed dog demonstrating dual CD3 and CD20 expression, with PCR for Antigen Receptor Rearrangement (PARR) revealing clonal rearrangements in both T-cell Receptor gamma (TCRγ) and immunoglobulin (Ig) heavy chain loci, suggesting a cross-lineage rearrangement. Similar molecular profiles have been documented in enteropathy-associated T-cell lymphomas (EATL), where neoplastic cells expressing both markers, with molecular analyses demonstrated monoclonal TCRγ rearrangements, indicating aberrant or cross-lineage antigen expression as a defining characteristic (6, 8, 12). Cutaneous epitheliotropic T-cell lymphoma (CETCL) with CD3 and CD20 co-expression has also been documented in dogs. Brachelente et al. (5) employed dual immunolabeling to confirm co-expression within individual neoplastic cells, with PARR analysis demonstrating monoclonal TCRγ rearrangement, supporting T-cell origin despite B-cell marker expression. Ewing et al. (13) further reported increased CD20 expression prevalence in canine CETCL, highlighting its potential diagnostic utility, though its prognostic significance remains controversial.

These findings collectively underscore the complexity of lymphoid differentiation and the limitations of the classification based solely on IHC phenotyping. Molecular diagnostics, particularly PARR, have provided crucial insights into these phenotypically complex cases, revealing concurrent clonal rearrangements of both TCRγ and Ig in selected cases, further challenging traditional classification paradigms (14, 15).

Despite these advances, the prevalence and the clinicopathological characteristics of these unusual phenotypes remain incompletely depicted. Here, we analyzed 33 cases of canine lymphoma using an integrated approach combining WHO-based morphological classification, comprehensive IHC immunophenotyping with T-cell (CD3 and CD5) and B-cell markers (CD20 and PAX5), and PARR analysis. By correlating these multiple parameters, we aimed to refine existing diagnostic frameworks, enhance lymphoma subtype classification, and address the challenges posed by phenotypically complex cases, particularly those demonstrating concurrent B- and T-cell marker expression.

## 2 Materials and methods

A retrospective analysis of the MyLAV Diagnostic Laboratory electronic database was conducted, examining canine cases diagnosed as “lymphoma” over a five-year period (1st June 2019 – 31st May 2024). From an initial cohort of 4.350 cases, 33 met the stringent inclusion criteria: (a) presumptive lymphoma diagnosis by European or American board-certified veterinary oncology specialists; (b) histopathological confirmation of a neoplastic round-cell infiltration; (c) histological review by a board-certified pathologist (GM); (d) standardized IHC analysis (CD3, CD5, CD20 and PAX5); (e) co-expression of minimum one T-cell and one B-cell marker on >80% neoplastic cells; and (f) comprehensive clonality testing for both TCR and immunoglobulin markers.

### 2.1 Histopathology

Tissue specimens were processed using standard protocols, including fixation in 10% neutral buffered formalin and paraffin embedding. Four-μm sections were prepared for hematoxylin and eosin (H&E) staining and evaluated according to the WHO classification criteria for canine lymphoma (2).

### 2.2 Immunohistochemistry

Immunohistochemical analysis was performed on serial paraffin sections using an automated immunostainer (Ventana Benchmark XT, Ventana Medical Systems Inc., Oro Valley, USA) following established protocols (16) and adhering to the American Association of Veterinary Diagnosticians Subcommittee guidelines for standardized IHC (17). The antibody panel included CD3, CD5, CD20 and PAX5 (Supplementary Table S1). Quality control measures incorporated reactive canine lymph node as positive control and equivalent-concentration anti-mouse IgG1 as negative control. Immunoreactivity was evaluated using a standardized scoring system. Distribution patterns were categorized as focal (less than 10–20% tissue positivity), multifocal (20–50% distributed positivity), or diffuse (more than 50% uniform positivity). Staining intensity was classified as low (faint immunoreactivity), moderate (clear but submaximal signal), or high (strong, uniform dark brown staining).

### 2.3 Molecular clonality analysis

PARR analysis targeted TCRγ, CBmajor and CBminor gene rearrangements. Genomic DNA was extracted from 3-μm deparaffinized sections using the QIAsymphony DSP DNA Mini Kit (Qiagen, Milan, Italy). PCR amplification was performed in duplicate 50-μL reactions, containing 25 μL of 2x HotStarTaq Master Mix (Qiagen), 0.3 μM of each primer (Supplementary Table S2), and 5 μL template DNA. Products were analyzed via capillary gel electrophoresis (QIAxcel Advanced System, QIAxcel DNA High Resolution Kit, Qiagen) and visualized using the QIAxcel ScreenGel Software 1.5 (Qiagen). PARR results were classified according to distinct patterns: clonal results demonstrated one or two distinct peaks with minimal polyclonal background; polyclonal patterns showed distributed fragments across the expected size range; pseudoclonal results exhibited variable peak sizes in duplicate assays; and negative results indicated the absence of amplification products within the target range.

## 3 Results

A total of 33 dogs with dual T- and B-cell marker expression (double-positive) lymphoma were identified in the MYLAV database in the 5-years period. The cohort included 7 (21.5%) crossbreeds, 7 (21.5%) Labrador Retrievers, 2 (6.1%) English Bulldogs, 2 (6.1%) English Cocker Spaniel, 2 (6.1%) French Bulldogs, 2 (6.1%) Maltese, and 2 (6.1%) Jack Russell Terriers. Additionally, a single case (3.0%) was recorded for each of the following breeds: Akita Inu, Beagle, Boxer, Dachshund, English Setter, Flat-Coated Retriever, Rottweiler and Shi-tzu. Breed information was unavailable for one dog. Males (19/33, 57.6%) outnumbered females (14/33, 42.4%), with all males being intact and all females spayed. The mean age at diagnosis was 9.6 ± 3.0 years, with a median of 10 years (range, 2–16 years).

The skin, oral/nasal mucosa and mucocutaneous junction were the most commonly affected sites, accounting for 24 cases (72.7%). Among them, 11 cases (45.8%) displayed epitheliotropism. The remaining cases involved the duodenum (4 cases, 12.1%), lymph nodes (2 cases, 6.1%), spleen (1 case, 3.0%), kidney (1 case, 3.0%), and pharynx (1 case, 3.0%). Among the 4 cases of intestinal lymphoma, full-thickness biopsies were available in 2, confirming a diagnosis of transmural medium-to-large cell lymphoma. In the other 2 cases, only endoscopic biopsies were available, leading to a diagnosis of mucosal small-to-medium cells lymphoma. Nodal and splenic cases were characterized as diffuse medium-to-large cell lymphoma. In the renal case, large lymphoid cells replaced the parenchyma and infiltrated the perirenal adipose tissue. Finally, the pharyngeal mass was diagnosed as large cell lymphoma.

**Figure 1:**
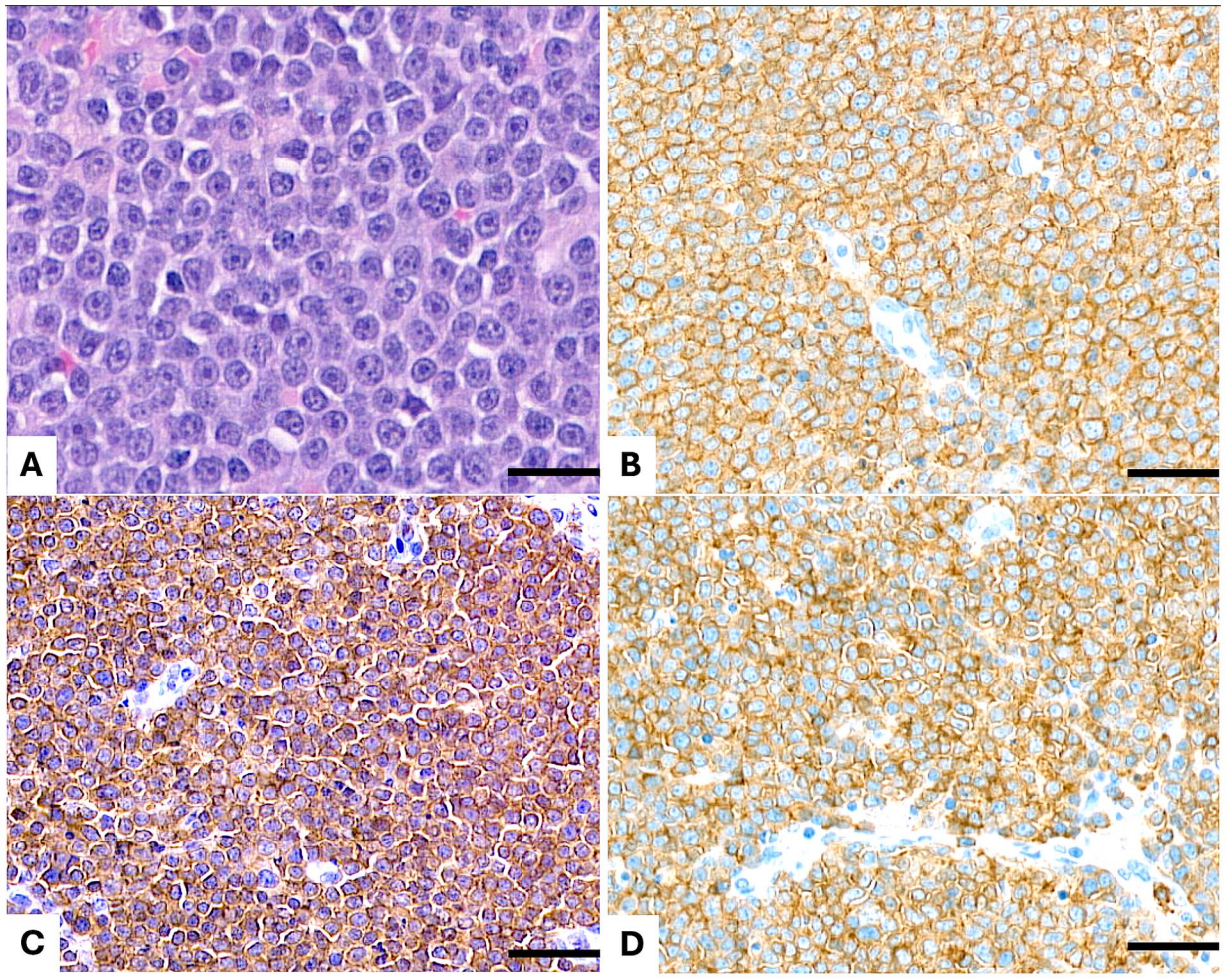
Lymph node. **(A)** Hematoxylin and eosin (H&E) staining reveals a diffuse, medium- to large-cell lymphoma composed of neoplastic cells with lightly dispersed chromatin, one single nucleolus, slightly irregular nuclei, and abundant cytoplasm. **(B)** Immunohistochemical staining for CD20 demonstrates diffuse membranous positivity in the neoplastic cells. **(C)** CD3 immunostaining shows diffuse membranous and cytoplasmic positivity in the neoplastic population. **(D)** CD5 immunostaining reveals diffuse membranous and cytoplasmic positivity in the neoplastic cells. Scale bar = 50 μm.

**Figure 2:**
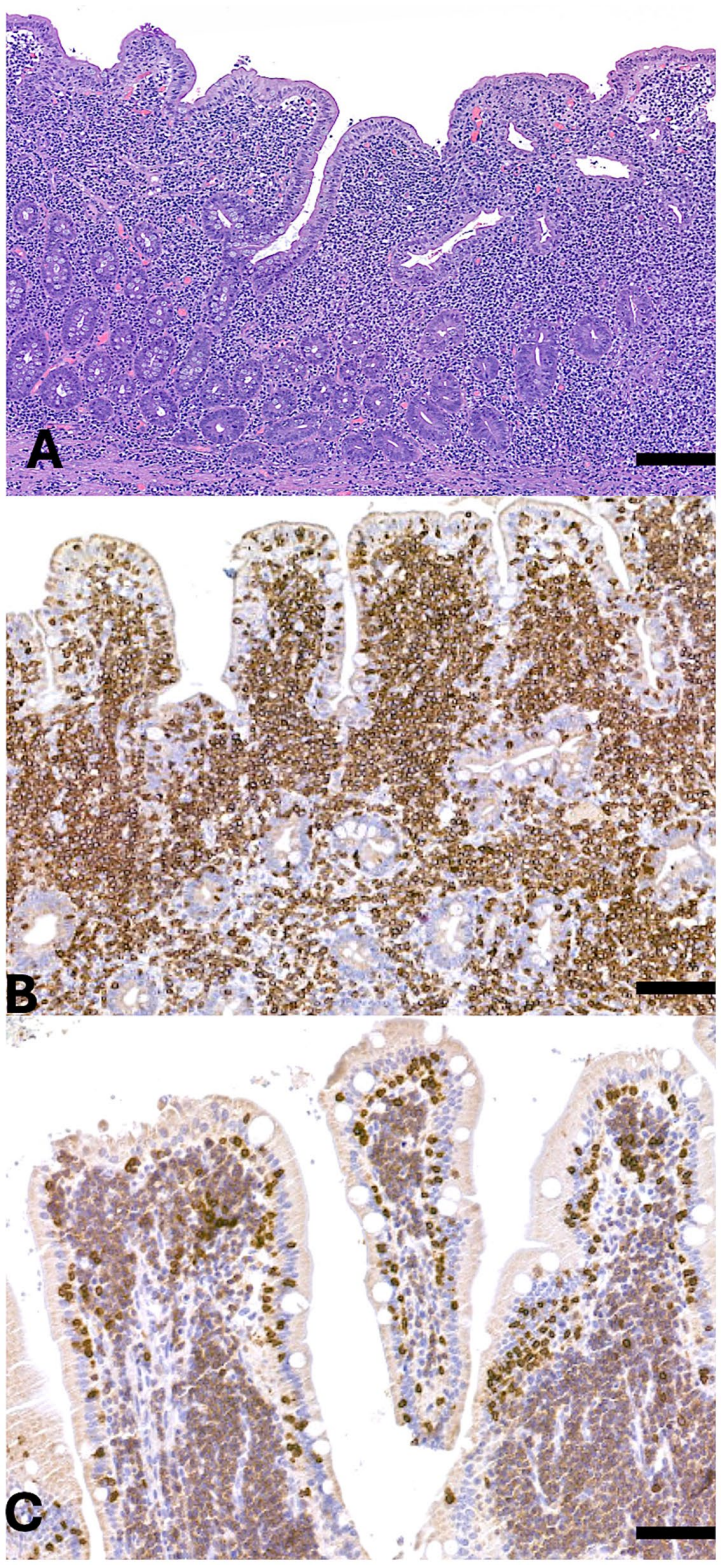
Small intestine. **(A)** Hematoxylin and eosin (H&E) staining of the mucosa and submucosa reveals diffuse infiltration by a population of medium-sized neoplastic cells extending into both the villi and submucosa. **(B)** Immunohistochemical staining for CD5 shows strong and diffuse membrane and cytoplasmic positivity in the neoplastic cells. **(C)** Immunohistochemical staining for CD20 demonstrates diffuse membrane positivity in the neoplastic population. Scale bar = 300 μm.

**Figure 3:**
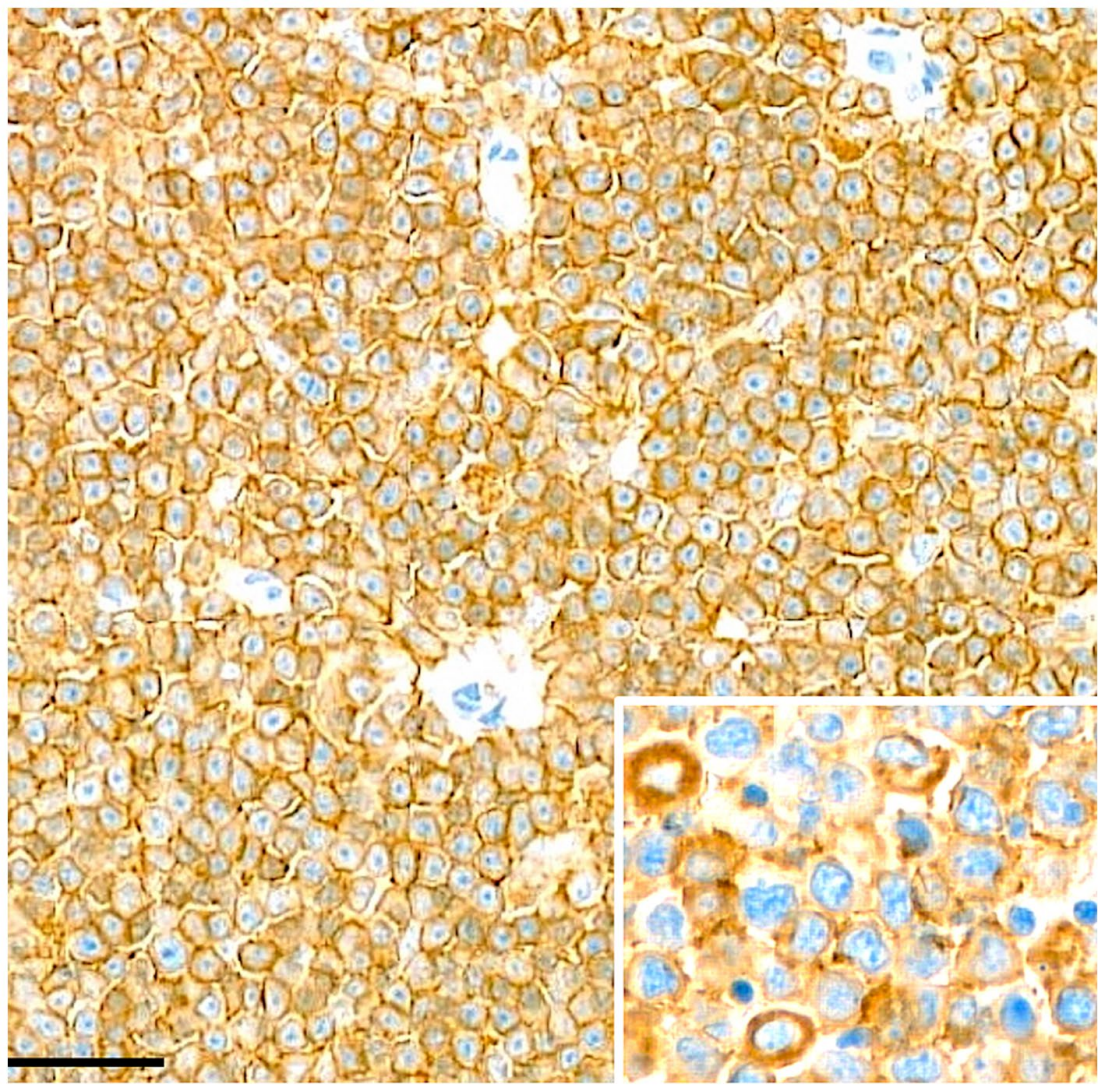
Lymph node. Immunohistochemical staining for CD20 demonstrates diffuse and cytoplasmic membranous positivity in the neoplastic cells. Scale bar = 50 μm.

Immunohistochemical analysis showed CD3 positivity in all cases, with 32/33 cases (97.0%) exhibiting both membrane and cytoplasmic expression. One cutaneous lymphoma case (3.0%) affecting the scrotal region showed CD3 expression limited to the membrane. Membrane CD3 positivity was intense and diffuse in 31 cases (93.9%), while 2 cases (6.1%) displayed moderate and multifocal positivity. Cytoplasmic CD3 expression was intense and diffuse in 27 cases (84.4%), moderate and multifocal in 3 cases (9.4%), and intense but multifocal in 1 case (3.1%). For CD5, 30 cases (90.9%) showed positive membrane expression, with 24 cases (75.0%) displaying intense and diffuse positivity, 4 cases (12.5%) showing intense and multifocal positivity, and 2 cases (6.3%) exhibiting moderate multifocal positivity. One case lacked CD5 reactivity in the IHC analysis. All cases showed CD20 membrane expression, with 29 (87.9%) cases also exhibiting cytoplasmic positivity of varying intensity. The staining patterns included intense and diffuse positivity in 19 cases (57.6%), moderate and diffuse positivity in 7 cases (21.2%), moderate and multifocal expression in 4 cases (12.1%), and intense and multifocal positivity in 3 cases (9.1%). PAX-5 nuclear expression was detected in 12 of 33 cases (36.4%), with 9 cases (27.3%) showing moderate and multifocal positivity, 2 cases (6.1%) showing occasional positive neoplastic cells, and 1 case (3.0%) displaying intense and diffuse positivity. Figures 1–3 illustrate the most characteristic morphological and immunohistochemical features of the cases presented in this study.

Molecular analysis revealed TCR gene rearrangement signals in 23 of 33 cases (69.7%), comprising 15 cases (45.5%) with pseudoclonal rearrangement and 7 cases (21.2%) with clonal rearrangement. CBmajor and CBminor signals were less common, with 4 dogs (12.1%) showing pseudoclonal rearrangement of CBmajor alone, two dogs (6.1%) showing pseudoclonal rearrangement of CBminor alone, and two cases (6.1%) displaying pseudoclonal rearrangement of both genes. One case (3.0%) showed a polyclonal result for CBmajor alone. No clonal rearrangements were identified for either CBmajor or CBminor. Among the 9 dogs with detectable CBmajor or CBminor signals, 7 (77.8%) showed concurrent TCR signals, including 6 cases with pseudoclonal patterns and 1 case with a clonal pattern. Individual case details for immunohistochemistry and PARR results were documented in Supplementary Table S3.

## 4 Discussion

This study analyzed 33 cases of canine double-positive lymphoma, providing comprehensive characterization of their histopathological features, IHC profiles, and clonality patterns. The predominant anatomical sites included cutaneous tissues, oral and nasal mucosa, and mucocutaneous junctions, with epitheliotropism observed in 46% of cases. This predilection for epithelial tissues aligns with previous findings in cutaneous and mucosal lymphomas, where tissue tropism might be mediated by specific interactions with the local immune microenvironment (18). Additionally, neoplastic involvement was documented in intestinal tissues, lymph nodes, spleen, kidneys, and pharynx, highlighting the diverse anatomical distribution patterns of these lymphomas. Histologically, the neoplastic population was mainly characterized by medium-to-large lymphoid cells, with notable morphological heterogeneity, particularly in intestinal cases. Nodal, splenic, and renal lymphomas predominantly displayed a diffuse large-cell pattern, suggesting an aggressive biological behavior (1). In one case, extensive infiltration of large lymphoid cells into the renal parenchyma and perirenal adipose tissue indicated advanced-stage disease with potential prognostic implications.

Immunophenotypic characterization confirmed concurrent CD3 and CD20 expression in all cases, supporting the classification of these lymphomas as a distinct pathological entity. Strong and diffuse CD3 immunoreactivity was observed in 94% of cases, consistent with previous reports indicating T-cell lineage predominance in these lymphomas (6, 15). CD20 expression was widely distributed and always co-expressed with CD3 + positive cells but demonstrated variable staining intensity. Notably, CD5 expression was detected in 94% of cases, with diffuse and intense immunoreactivity in 75%, suggesting phenotypic similarities with conventional T-cell neoplasms, as CD5 represents a well-established marker of mature T-cell malignancies (1, 19). PAX5, a critical B-cell lineage-specific transcription factor, was detected in only 36% of cases. This master regulator plays an essential role in B-cell identity through modulation of lineage-specific gene expression programs. Its variable expression in double-positive lymphomas suggests either partial lineage plasticity or selective PAX5 downregulation. The limited presence of PAX5, typically expressed in mature B-cell and select precursor B-cell malignancies, indicates a restricted role in lineage determination within this distinct entity (20).

Aberrant cytoplasmic CD20 expression was frequently observed in this study. While CD20 is traditionally characterized as a membrane-bound B-cell marker mediating cellular activation and differentiation in both humans and dogs, cytoplasmic CD20 expression has previously been documented only in human T-cell lymphomas (21). This unusual pattern raises fundamental questions about the underlying molecular mechanisms. Several hypotheses may explain this phenomenon. One possibility suggests these lymphomas arise from a distinct subset of T-cells that constitutively express CD20. Although CD20 expression is typically absent in T-cells, rare CD20-positive T-cell populations have been identified in both physiological and pathological human conditions (22, 23). Supporting this hypothesis, recent findings in dogs have demonstrated a T-cell subpopulation expressing B-cell markers in non-neoplastic states (24). Alternative mechanisms involve genetic or epigenetic dysregulation that may disrupt lineage-specific gene expression patterns, resulting in ectopic CD20 expression within malignant T-cells (25, 26).

The presence of CD20 in CD3-positive lymphomas carries significant implications for both diagnosis and therapeutic intervention. From a diagnostic perspective, CD20 expression could potentially lead to misclassification as a B-cell malignancy if comprehensive T-cell marker analysis is not performed (22, 23). Therapeutically, the clinical relevance of CD20 expression is contingent upon its subcellular localization. Surface expression of CD20 may confer susceptibility to B-cell-directed therapies, including APAVAC, whereas restricted cytoplasmic localization may diminish therapeutic efficacy (27, 28).

PARR analysis revealed clonal or pseudoclonal TCR rearrangements in 70% of cases, confirming a predominant T-cell origin despite CD20 co-expression. Conversely, CBmajor and CBminor alterations were detected at substantially lower frequencies (12 and 6% of cases, respectively), with no evidence of clonality. These molecular findings corroborate previous studies demonstrating that double-positive lymphomas maintain a dominant T-cell molecular signature despite their mixed immunophenotype (13).

However, interpreting PARR results can be challenging due to the potential for false positive and false negative results. False positives may arise from the amplification of clonally expanded, but non-neoplastic, lymphocytes present in reactive or inflammatory conditions, leading to an overestimation of clonality. Additionally, pseudoclonality can occur due to preferential primer annealing or limited polyclonal template diversity in cases with low cellularity, which may artificially skew results. False negatives, on the other hand, are often due to incomplete primer coverage, where certain V-J recombinations are not detected because of a limited or suboptimal primer set. This issue is particularly relevant given the high degree of genetic diversity in antigen receptor loci, which varies between species and even between individuals. Furthermore, somatic mutations or deletions affecting primer-binding sites can prevent efficient amplification, leading to an underestimation of clonal populations. In some cases, tumors with extensive genomic instability may exhibit large-scale rearrangements that disrupt the typical amplification targets, further complicating molecular detection (29). In our study, the detection of pseudoclonal Ig rearrangements in a subset of cases suggests either incomplete V(D)J recombination events or secondary genetic alterations affecting antigen receptor loci, further illustrating the molecular complexity of lymphoid malignancies. Significantly, concurrent TCR and Ig rearrangements were identified in only seven cases, supporting the hypothesis that these represent cross-lineage mechanisms rather than true biphenotypic differentiation. This phenomenon emphasizes that antigen expression patterns alone may not definitively establish lineage commitment in these lymphomas. Notably, a subset of cases showed no detectable TCR or Ig gene rearrangements by PARR analysis. This genetic profile could be attributed to several factors: the presence of somatic mutations affecting primer binding sites, the emergence of rare V-J combinations not covered by standard primer sets, or the possibility that these lymphomas arise from very early lymphoid precursors prior to antigen receptor gene modification (30, 31).

Several limitations of this study warrant consideration. While the sample size of 33 cases is notable given the relative rarity of double-positive lymphomas, it may constrain the broader applicability of these findings to the general population. A significant limitation is the absence of therapeutic intervention data and longitudinal follow-up, which precludes analysis of treatment responses and prognostic outcomes. Understanding the clinical behavior and therapeutic response of these lymphomas would provide valuable insights for optimizing patient management protocols. Future investigations incorporating larger patient cohorts and comprehensive follow-up data will be essential for refining both the classification criteria and prognostic indicators for this disease entity. In conclusion, this investigation contributes significantly to the expanding knowledge base regarding canine double-positive lymphomas, particularly in elucidating their complex immunophenotypic profiles, clonal pattern and anatomical distribution. The findings emphasize that while IHC remains a fundamental diagnostic tool, it may be insufficient in isolation for definitive lineage determination in these cases. PARR analysis emerges as an essential complementary technique for distinguishing between aberrant marker expression and true biphenotypic differentiation.

## Data Availability

The original contributions presented in the study are included in the article/Supplementary material, further inquiries can be directed to the corresponding author.
