# Supplementary material for: Morphological, phenotypical and molecular characterization of canine lymphomas with dual T- and B-cell markers expression
Source: Front Vet Sci. 2025 Apr 22;12:1578425. doi: 10.3389/fvets.2025.1578425 (PMC12053485; doi:10.3389/fvets.2025.1578425)
Supplement: Supplementary file 1 [file Table_1.DOCX]

Supplementary Material

**SUPPLEMENTARY TABLE 1** details of antibodies used for immunohistochemical analysis of 33 cases of canine double-positive lymphoma

| **Target** | **Specificity** | **Clone** | **Source** |
| --- | --- | --- | --- |
| CD3 | T-cells | F7.2.38, monoclonal | Dako, Glostrup, Denmark |
| CD5 | T-cells | SP19, monoclonal | Roche Diagnostics, Monza, Italy |
| CD20 | B-cells | SP32, monoclonal | Abcam, Cambridge, UK |
| PAX5 | B-cells | SP34, monoclonal | Roche Diagnostics, Monza, Italy |
