# Supplementary material for: Morphological, phenotypical and molecular characterization of canine lymphomas with dual T- and B-cell markers expression
Source: Front Vet Sci. 2025 Apr 22;12:1578425. doi: 10.3389/fvets.2025.1578425 (PMC12053485; doi:10.3389/fvets.2025.1578425)
Supplement: Supplementary file 2 [file Table_2.DOCX]

Supplementary Material

**SUPPLEMENTARY TABLE 2** details of primer sets used for PCR for Antigen Receptor Rearrangement (PARR) analysis in 33 cases of canine double-positive lymphoma

| **Receptor** | **Primer Sequence*** |
| --- | --- |
| B-Cell Receptor (BCR) | 5'-TTCCCCCTCATCACCTGTGA-3'  5'-GGTTGTTGATTGCACTGAGG-3'  5'-CAGCCTGAGAGCCGAGGACAC-3'  5'- TGAGGAGACGGTGACCAGGGT-3'  5'-CAGCCTGAGAGCCGAGGACAC-3'  5'-TGAGGACACAAAGAGTGAGG-3' |
| T-Cell Receptor (TCR) | 5'-ACCCTGAGAATTGTGCCAGG-3'  5'-GTTACTATAAACCTGGTAAC-3'  5'-TCTGGGVTGTAVTACTGTGCTGTCTGG-3' |

*Reference: Burnett RC, Vernau W, Modiano JF, et al. Diagnosis of canine lymphoid neoplasia using clonal rearrangements of antigen receptor genes. Vet Pathol 2033;40(1):32-41
